# To Live Together Is to Move Together: Social Actigraphy Applied to Healthy Elderly People

**DOI:** 10.3390/s22166011

**Published:** 2022-08-11

**Authors:** Marco Rabuffetti, Ennio De Giovannini, Maurizio Ferrarin

**Affiliations:** 1 IRCCS Fondazione Don Carlo Gnocchi, 20148 Milano, MI, Italy; 2 Centro Medico Riabilita Cooperativa Sociale Mano Amica Onlus, 36015 Schio, VI, Italy

**Keywords:** social actigraphy, dyad, marital couple, accelerometer, correlation, rehabilitation

## Abstract

(1) Background: Actigraphic methods allow prolonged monitoring of human physical activity (PA) by wearable sensors in a real-life unsupervised context. They generally do not characterize the social context, and nearby persons can have a modulating effect on the performed PA. The present study aims to apply an existing method for bimanual actigraphy to both components of a marital dyad to verify the level of association between the two PA profiles. Other dyad comparisons complete the overall figure. (2) Methods: Seven-day actigraphic recordings collected from both components of 20 married couples of retired, cohabiting, healthy subjects (age ranging from 58 to 87 years) were considered. (3) Results: PA profiles of a marital dyad are significantly more correlated (coefficient: 0.444) than unrelated couples (0.278). Interestingly, participants’ profiles compared with their own recording shifted by 24 h, evidencing an intermediate level of association (0.335). Data from the literature, the high association (0.875) of individual right and left wrist profiles, enforce the analysis. (4) Conclusions: The proposed method, called “social actigraphy”, confirmed that the partner has a relevant effect on one’s PA profile, thus suggesting involving the partner in programs concerning lifestyle changes and patient rehabilitation.

## 1. Introduction

Moving together in a coordinated manner is often observed in animals. Insect swarms, fishes, bird flocks, and herbivore herds are clear examples of the advantages of coordinated moving in a group compared to individual moving; therefore, such social behaviors have been favored by evolution [1,2]. Almost-perfect synchronized movement can be observed, such as in coordinated mating rituals, but usually, these coordinated behaviors are reciprocally associated with time shifts, e.g., several seconds up to several minutes. In addition, humans often move together in response to social constraints (school and work timetables, store openings, etc.), as well as to implicit suggestions from those living close by [3,4]. While CNS theory has studied the concurrent movement of the upper limbs performed by two subjects face to face, evidencing a coupling effect [5], it has been proposed that humans tend to also associate their complex behaviors in real life with unsupervised conditions [6]. This association has been verified proactively in parent–child dyads [7] and has been underlined as a facilitating factor in potentially increasing physical activity, generally associated with better health [8].

Such PA associations in unsupervised contexts and in real life have been detected by questionnaire approaches [9] and, in some studies, by objective activity tracking methods, generally referred to as actigraphy, based on wearable sensors [10,11,12]. Harada [10] demonstrated by accelerometric 7-day monitoring that daily sedentary behaviors were fairly associated (beta = 0.30–0.47) between 72 marital dyad components. Interestingly, this was apparently not influenced by the degree of reciprocal attachment.

Ashe [11] confirmed by 7-day accelerometry that daily sedentary behaviors were correlated between 112 familiar dyad components (r = 0.44). Pauly advanced the methodological approach by assessing intra-dyad covariations of 7-day accelerometry on an hourly rather than daily basis, thus getting closer to the concept of synchrony [12]. Significant results from 414 couples pooled from two different studies showed moderate to vigorous physical activity (MVPA) synchrony ranging from 0.35 to 0.42 and sedentary synchrony ranging from 0.36 to 0.39.

These recent studies quantify an association between lifestyle motor behaviors. Even the reference to “moving in sync” in a recent study [12] is more related to an agreement between lifestyles than to a substantial synchronization of behaviors, which would require a much shorter epoch definition than the 1 h epoch approach used in that study. The present study, and particularly its methodology, aims to provide a more focused glimpse at the phenomena of the association between motor behaviors in dyads. This is obtained by reducing the time detail of the method to 1 min epochs. We believe that this novel, more focused approach can provide a more robust basis for the concept of association between physical activity profiles in the dyad of persons living together and, therefore, provide a more solid basis for the identification of an association that could possibly be involved in therapeutic intervention, with one component of the dyad “towing” the other.

The objectives of the present retrospective study were:

1. To provide a clinically oriented method and a related sensitive and specific metric for between-subject PA association, advancing the previously published approaches;
2. To quantify the PA association in actigraphic recordings collected in healthy elderly couples living together, comparing this information with other applicable conditions (such as persons not living together).

## 2. Materials and Methods

### 2.1. Participants

Participants and related data were selected from a larger collection of actigraphic recordings and routinely managed to build a normative data set able to support the clinical actigraphic assessment of patients with Parkinson’s disease. The inclusion criteria were: be 50 years old or above, have a healthy condition, belong to a couple whose components are both retired, and have concurrent recordings of both components of the couple. Therefore, 40 healthy individuals (20 females, age range: 58–83, mean: 71 years old; 20 males, age range: 59–87, mean: 73 years old) from 20 married cohabiting couples (the considered dyads) were included in the present study. All subjects had already retired from their jobs, and their daily activities were not necessarily forcing them to leave home and part from the partner, which, in our approach, is the complementary component of the dyad. All participants were Italian and living in the area north of Vicenza (Italy), and all dyads anecdotally reported an active lifestyle involving frequent activities in the nearby wildlife areas. Data were collected in the frame of the clinical activity of coauthor E.D.G., in accordance with the ethical standards of the responsible committee on human experimentation (institutional and national) and with the 1975 Declaration of Helsinki, as revised in 2000, and all the participants provided signed informed consent to participate in the study and allow their data to be analyzed for and presented in an anonymous format for research objectives.

### 2.2. Data Collection and Analysis

All subjects wore a wearable actigraph (GENEActiv, Activinsights, Huntingdon, UK) on their nondominant wrist. They were asked to wear the device 24/7 without removing it, except for discomfort or health-related issues. The device was waterproof and compatible with water and hygiene-related activities. The recording could start at any time in the day, and it was synchronized between the two individuals of each dyad. The individuals provided informed consent prior to the beginning of the 7-day monitoring period. Data collection took place between February 2019 and February 2020 (36 out of 40 participants), and, due to COVID restrictions being lifted in summer 2020, the last 4 participants were recorded in July 2020.

**Figure 1:**
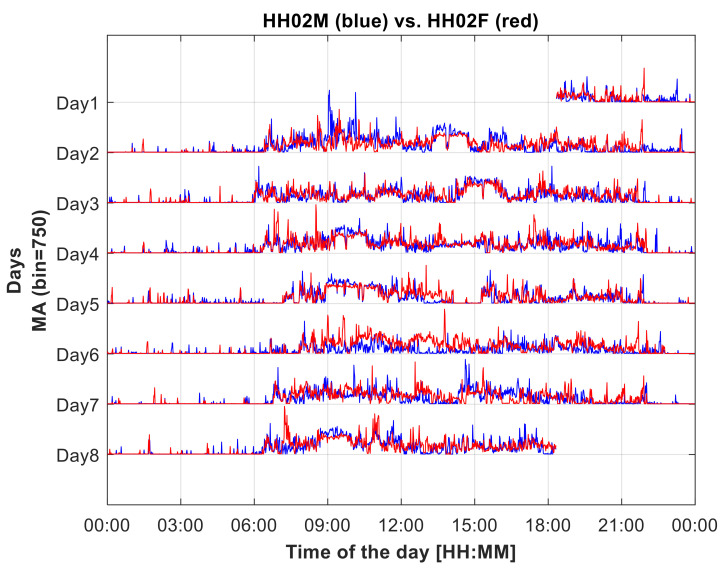
Synchronized plots of the week-long MA profiles of the two subjects (HH02M, male, in blue, and HH02F, woman, in red) belonging to a married couple. The CC value between them is 0.58, the highest value observed in the study, marking a relevant coupling, noticeable in the common wake-up time, frequent phases of common MVPA, and common sleeping time.

The acceleration associated with the wrist was measured at a 100 Hz sampling rate by a triaxial accelerometer embedded in the actigraph, stored onboard, and downloaded after the conclusion of the recording. Subsequent analyses followed the method described in [13], which was originally developed to quantify the association between motor activity profiles in both wrists, in order to study handedness or lateral prevalence in hemisyndromes. The first step of the method consisted of an epoch-based motor activity profile quantification. The epoch duration was set to 1 m [14]. Therefore, the raw individual data set consisted of 7 (days) × 1440 (epoch/day) = 10,080 values of the motor activity (MA) index computed in 1 min epochs (example of synchronized data set from a dyad in Figure 1). The single value of the MA index MA_e_ consists of the standard deviation of the acceleration vector magnitude in the considered 1 min epoch, implemented in the following formula [13], where *a_j_* is the norm for acceleration triplet measured at time *j* (*n* values inside epoch *e*)

$$MA_{e}=\sqrt{\frac{\sum_{j=1}^{n}(a_{j}-\frac{1}{n}\cdot \sum_{j=1}^{n}a_{j}{)}^{2}}{n-1}}$$

According to the method for studying the association between the motor activity of the right and left upper limbs [13], the association between two MA profiles was quantified by the correlation coefficient (CC, as obtained by the “corrcoeff” MATLAB function applied to the MA profiles, with zero lag) between the two profiles (values ranging from 1, perfect synchrony, to 0, absence of any synchrony, to negative values, for inversely correlated profiles), as well as by the asymmetry ratio (AR), quantifying the prevalence in magnitude of one or the other profiles (values range from 100% to 100%, where 0% marks the equivalence in magnitude between sides). Reference data from an available dataset with a concurrent recording of right and left wrist actigraphic profiles [15] are here considered a term of comparison, since the right and left limbs of the same person generally have a fairly high association.

### 2.3. Statistical Analysis

The overall dataset consisted of 40 individual datasets, i.e., MA profiles, matched two by two according to the dyad and with information about individual sex.

By directly applying the method proposed in [13] to all possible combinations of the two MA profiles, either from the components of the dyad or between unrelated individuals, the following CC indexes (and implicitly also the AR indexes) were obtained:

- CC_couple_, intra-dyad CC, 20 values;
- CC_betweenXX_, extra-dyad CC between two individuals belonging to different dyads and of the same sex, 380 values;
- CC_betweenXY_, extra-dyad CC between two individuals belonging to different dyads and of complementary sex, 380 values.

Moreover, when comparing any single MA profile with itself being shifted by 24 h and 12 h, respectively, the following indexes were obtained by considering the overlapped sections, 6 days and 6 days and a half, respectively:

- CC_self24_, intrapersonal CC (comparing an individual MA profile with the 24-h-shifted self, computed on the 6-day overlapped profile sections), 40 values;
- CC_self12_, unrelated intrapersonal CC (comparing an individual MA profile with the 12-h-shifted self, computed on the 6 and a half day overlapped profile sections), 40 values.

Additionally, a different data set from a Master’s thesis (26 healthy adult subjects wearing sensors on both wrists for 24 h) was considered to quantify the association between the right and left upper limbs [15]:

- CC_RxLx_

The adopted method also produced an additional index, the asymmetry ratio (AR) between the two considered motor profiles, which was introduced to quantify hand dominance [13] and quantify the severity of the hemilateral motor syndromes in the upper limbs of a person who suffered unilateral brain damage [16]. Here, when comparing different individuals, the absolute value of the AR quantifies the attitude of one dyad component to be comparatively more or less active than the other.

The CC table of the overall results was analyzed by nonparametric tests, an analysis of variance (Kruskal–Wallis), followed by post-hoc multiple comparisons (Wilcoxon–Mann–Whitney). The *p*-value was set to 0.05, and in the multiple comparisons, the *p*-value was corrected with the Bonferroni–Holm’s method to minimize the effect of multiple comparisons.

## 3. Results

In Figure 1, the MA profiles of the two components of a couple are plotted superimposed. Anecdotally, for example, on Day 2 at about 1:00 to 3:00 p.m. or on Day 3 at about 2:00 to 4:00 p.m. and so on, the coupling between the two profiles is relevant to the unaided eye. Likewise, on Day 6 at about 1:00 to 2:00 p.m., the two profiles profoundly differ.

**Figure 2:**
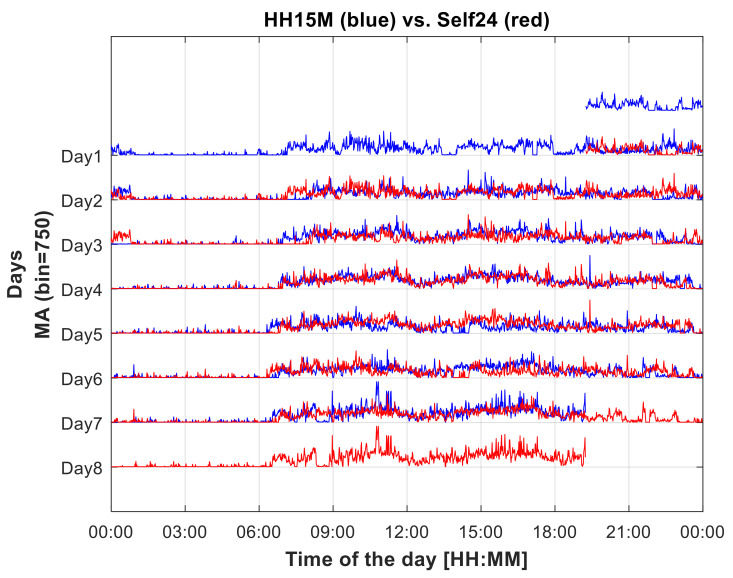
Plots of a week-long MA profile (HH15M, male, in blue) along with the 24-h shifted self (in red), i.e., the same MA profile shifted by 24 h, in order to show the best CC_self24_ (value: 0.50), the best association between an individual profile, and its 24 shifted one. The subject had a very regular daily timeline and habits, which led to a strong agreement between different days. CC_self24_ is computed on a 6-day overlap section of the profiles.

As another example, an individual MA profile is presented in Figure 2 along with its self-time displaced by a 24 h lag. Noticeably, a regular daily schedule is a major determinant in producing a high CC_self24_ coefficient.

**Figure 3:**
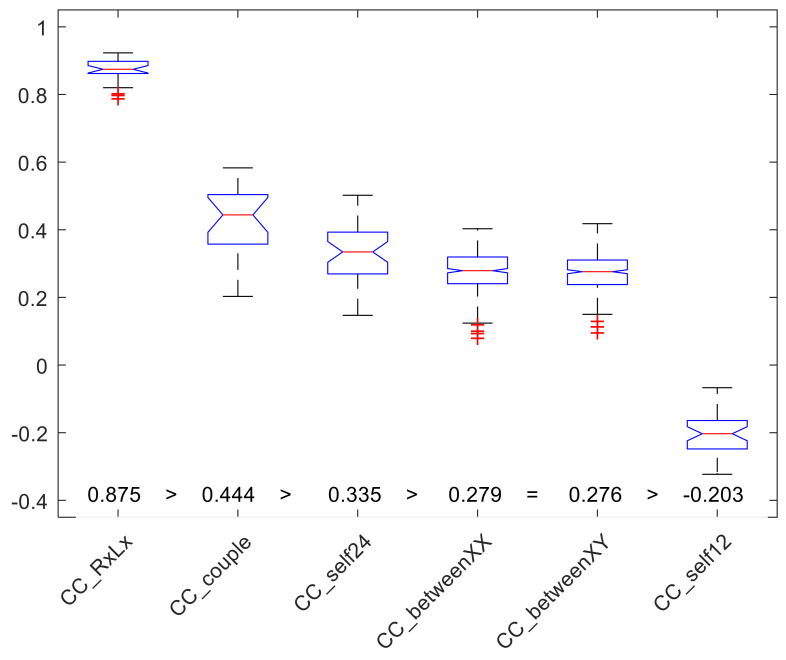
Bar plots of the CC values grouped according to the conditions considered (plotted values include median, reported also as numerical values, first and third quartiles, nonoutlier extremes, and, where present, outliers as “+”). The statistical analysis (Kruskal–Wallis, ANOVA, and Wilcoxon–Mann–Whitney post-hoc tests) evidenced that all conditions have different values compared to the other groups (the only exception is that CC_betweenXX_ and CC_betweenXY_ did not show different values). Median values are reported just above the *x*-axis labels, with significant differences between groups. The CC_RxLx_ group is presented as a reference, derived from a different data set published elsewhere [15].

The CC indexes computed, according to the presented analysis design, on the whole dataset are summarized as box plots in Figure 3. Additionally, as a term of reference, the same CC index is reported in the box and whiskers on the extreme left, as obtained by considering concurrent recordings at the right and left wrists, derived from a handedness study presented elsewhere [15]. Noticeably, as expected, this latter condition shows higher CC values.

Interestingly, CC_couple_ has the largest value among the combinations considered in the present study, underlining the fact that to live together is to move together. Just below that, CC_self24_ shows how personal habits concerning the daily schedule play a relevant role. Both CC_between_ indexes, while still marking some alignment between behaviors, have lower values. The reason for these relatively low values is the fact that almost all humans (and all our participants) tend to wake up early in the morning, have an active morning, relax in the midday, and reactivate in the afternoon until the evening, where humans prepare themselves for their nightly sleep. This overall scheme is obviously not realized within the 1 min precision, and this explains the relatively low values of the CC_between_ indexes. Finally, the CC_self12_ low and negative value simply states that night and day are characterized by opposite behaviors and that circadian rhythms are organized on a 24 h rather than a 12 h cycle.

The AR index values (not reported) computed at the same time as the CC index showed large variability and no statistical difference between the different conditions; therefore, there are no differences in the dyad asymmetries observed inside a marital dyad and in unrelated dyads.

## 4. Discussion

The association between motor activities was quantified in the present article by considering the correlation between two time profiles of physical activity, as measured by a wearable accelerometer on the nondominant wrist and summarized by the motor activity (MA) index. This method had already been developed and applied by the same research group to compare the motor behavior of the right and left upper limbs, to study hand dominance in healthy subjects [13] and asymmetry in the movement of upper limbs in patients with unilateral brain damage [16]. The time profiles of MA were computed for 1 min epochs [14], in order to provide high time details and a good approximation to the concept of synchronization [12]. This latter aspect represents the novelty of the study and an advancement relative to the larger time windows of 1 h [12] or even 1 day [10,11] adopted by previously published studies.

The participants were selected from a dataset of healthy subjects collected to support clinical routine, married and living together, older than 50 years, and retired from their job. This ensured that it was reasonable to seek a coupling of their daily activities. Interestingly, the design of the present study allowed computing a series of correlation coefficients, in addition to the basic coefficient concerning the couple interaction: the association between the motor activities profiles of two individuals not living together and of one individual vs. themselves 24 h and 12 h apart. These indexes are expected to provide, for the between index, reference values concerning a generic association expected between two individuals sharing all the social constraints concerning timetables, in addition to sharing a common geographic area, a common culture, and a common welfare. For the 24 h shift index, reference values concerning the individual relevance of consistent personal daily habits are, for the 12 h shift index, the inferior limit expected for an association index, since it is commonly accepted that switching night for the day depicts a completely different lifestyle. Moreover, an already-available dataset concerning handedness in bimanual actigraphy [15] was included as an upper limit for the computed CC association index.

Potentially confounding extrinsic factors are the time of year of the recording week, with expected differences in daylight hours and average weather conditions. Moreover, instantaneous weather conditions can be a confounding factor even for closely related recording weeks or days. All of these factors are expected to worsen the CC association index; therefore, we can expect a quota of underestimation in the CC between profiles obtained in different weeks. Nonetheless, since, in the modern way of life, the relevance of weather or daylight has been greatly reduced compared to the old days of preindustrial civilization, these confounding factors might play a lesser role than we expect.

While the CC association index quantifies how similar in form the considered motor activity profiles are, the AR index quantifies the prevalence in average intensity of one of the components of the dyad. This number is not that relevant in the present study involving only healthy adult subjects, but it will become relevant once asymmetric dyads are considered (for example, a person with a neuromotor disease and their healthy partner). We look forward to considering this index a quantification of the ability of one of the dyad components to tow, or conversely slow down, the other component.

The results confirmed the hypothesized major effect: those who live together move together. This hypothesis is supported either by the absolute value of the intra-dyad correlation coefficient, CC_couple_, having values between 0.203 and 0.583 (median: 0.444) in extremely good agreement with Harada, Ashe, and Pauly’s articles, and by the relative ranking of that value compared to the association between Rx vs. Lx wrists (CC_RxLx_ range: 0.787–0.923) and the association between unrelated dyads (CC_between_ range: 0.079–0.418). Needless to say, the wrists of a person are a dyad that truly shares a high association between life habits, only slightly modulated by a few high-energy unimanual tasks. On the contrary, analysis of unrelated couples allowed by the experimental design provides a reference value quantifying a common lifestyle between all individuals belonging to a major social community sharing culture and external triggers. It is worth noting that no difference was observed between unrelated dyads of persons of the same sex compared with unrelated dyads of persons of different sex. This implies that in our sample, no sex bias concerning daily activities was present. Nonetheless, no generalizations are advanced here, and any hypothesis on sex-related bias needs to be explored in further studies.

It is worth noting that an individual, compared to themselves 24 h later (CC_self24_ range: 0.147–0.502), shows a lower association compared to that with a partner. Since we had no hypothesis about this comparison, we expected no significant differences. On the contrary, the association inherited from living together proved to be more effective in determining a stronger association, compared to personal habits, which are mainly responsible for associations between one’s physical activity profiles day after day. The comparison between an individual and themselves, 12 h apart, substantiated obvious evidence: healthy subjects generally do not have comparable behaviors 12 h apart, and the always-negative correlation index means that a person completely changes motor behavior, active vs. sleeping, and vice versa. Someone, fond of classical literature, could also possibly indulge in a scientific demonstration of the differences between Dr. Jekyll and his nightly self, Mr. Hyde.

Weather conditions are expected to influence outdoor activities and therefore have an impact on actigraphic recordings, but no extreme conditions were observed during the data collection (only 3 couples out of 20 had an average day temperature <10 °C, and 3 out of 20 had >10 mm/day of rainfall).

Since a cross-correlation analysis does not allow identifying causality effects but only associations, it is reasonable that causality might be reciprocal. This is expected in the present experiment involving only healthy subjects, but it is also reasonable in asymmetric dyads where a person with motor disturbances is matched with a healthy partner. In this case, a towing effect of the healthy individual is as possible as a braking effect by the patient.

It is also necessary to remember that while quantity of association is a relevant aspect, quality of association, possibly restrained to a few hours a day, might be relevant as well.

The retrospective design of the study implied a sampling bias: participants were all from a small region (north by Vicenza), active above average, and potentially classifiable not only as healthy elderly but more precisely as healthy active elderly.

Moreover, the analysis did not control possible covariant factors: day types were not considered because the difference between weekend and working days, which is a relevant factor for persons with a job, is less relevant for retired persons. Weather conditions were not controlled, so “between” and time-shifted analyses may compare activities performed under different weather conditions, though a survey of weather data showed the absence of extreme weather conditions.

## 5. Conclusions

Results of the present study showed a relevant association between motor activity time patterns during several days in cohabiting elderly married couples. It is possible to state that to live together is to move together. Although causality cannot be inferred, such as who attracts whom and who slows down whom, it can be assumed that causality may work bidirectionally. This opens up the possibility of a rehabilitation intervention aimed at stimulating and increasing the motor activity of a specific patient, by involving the cohabiting partner in increasing their motor activities. This is expected to also induce an increase in motor activity in the target patient [17,18].

Interestingly, the marital couple is not the only dyad possibly considered responsible for motor activity associations. Other dyads or multiple groups might work as well, including kids or pets.

Future studies will explore these possible occurrences.

## Data Availability

Samples of data can be obtained by contacting the corresponding author.
